# RBV: Read balance validator, a tool for prioritising copy number variations in germline conditions

**DOI:** 10.1038/s41598-019-53181-7

**Published:** 2019-11-15

**Authors:** Whitney Whitford, Klaus Lehnert, Russell G. Snell, Jessie C. Jacobsen

**Affiliations:** 1 School of Biological Sciences, The University of Auckland, Auckland, New Zealand; 2 Centre for Brain Research, The University of Auckland, Auckland, New Zealand

**Keywords:** Computational biology and bioinformatics, Genetics

## Abstract

The popularisation and decreased cost of genome resequencing has resulted in an increased use in molecular diagnostics. While there are a number of established and high quality bioinfomatic tools for identifying small genetic variants including single nucleotide variants and indels, currently there is no established standard for the detection of copy number variants (CNVs) from sequence data. The requirement for CNV detection from high throughput sequencing has resulted in the development of a large number of software packages. These tools typically utilise the sequence data characteristics: read depth, split reads, read pairs, and assembly-based techniques. However, the additional source of information from read balance (defined as relative proportion of reads of each allele at each position) has been underutilised in the existing applications. Here we present Read Balance Validator (RBV), a bioinformatic tool that uses read balance for prioritisation and validation of putative CNVs. The software simultaneously interrogates nominated regions for the presence of deletions or multiplications, and can differentiate larger CNVs from diploid regions. Additionally, the utility of RBV to test for inheritance of CNVs is demonstrated in this report. RBV is a CNV validation and prioritisation bioinformatic tool for both genome and exome sequencing available as a python package from https://github.com/whitneywhitford/RBV.

## Introduction

There are four main types of variation in the human genome: single nucleotide variants (SNVs), small-scale changes in genomic content in the form of short indels, structural variants, and aneuploidies. Structural variants consist of medium to large-scale changes to the genomic structure, and includes both balanced chromosomal rearrangements (such as inversions and translocations) and copy number variants (CNVs). CNVs are typically defined as deletions or multiplications of sections of the genome, resulting in changes of genomic content greater than 1 kb^1^. Initial efforts to map genetic variation on the whole genome scale indicated that SNVs constituted the majority of variation between individuals^2^. However, large scale collaborations mapping CNVs in the human genome found on average an individual harbours over 1,000 CNVs of 443 bp or greater^3–6^. Taken together, although there is a greater number of SNVs per individual (approximately 3.6 million or ~0.1% of the genome^5^), due to the greater average size of CNVs and indels, they are responsible for greater genomic variance between genomes (up to 48.8 Mb or ~1.5%^6^).

CNVs play an important role in gene expression with changes in genetic content larger than 1 Mb estimated to be responsible for 17.7% of the genetic impact on gene expression^7^. One would expect that the proportion of genetically controlled variation in gene expression attributable to CNVs would be higher if CNVs smaller than 1 Mb were included in such analyses. CNVs are able to affect gene expression directly through copy number changes of genes and regulatory elements^8^, and indirectly through unmasking of recessive alleles^9^ and positional effects^10^. As such, there has been an increasing volume of research into the role of CNVs in disease. In particular, CNVs have been implicated in the aetiology of neuropsychiatric disorders including schizophrenia, intellectual disability, and autism spectrum disorder (as reviewed by Malhotra & Sebat, 2012^11^). Therefore, chromosomal microarray (CMA) has become a first-tier clinical diagnostic test for patients with unexplained intellectual disability, autism spectrum disorder, or multiple congenital anomalies, with diagnostic yield of 15–20% (reviewed by Miller, *et al*.^12^). The use of high throughput sequencing (HTS) in the form of whole exome sequencing (WES) and whole genome sequencing (WGS) is increasing for diagnostic testing, both due to its decreasing cost and ability to investigate genetic variants without prior hypotheses. HTS based methods offer the potential of identifying SNVs, indels and CNVs (including those not detected by current diagnostic CMA thresholds^13^) in a single test.

**Figure 1:**
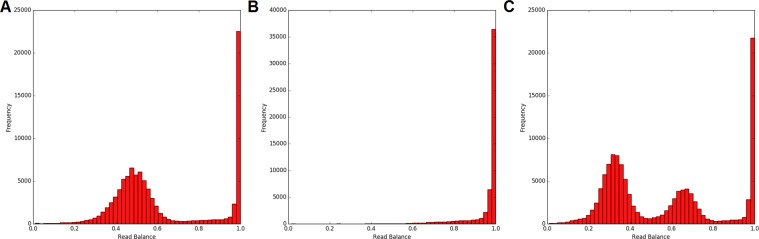
Distribution of relative reads for diploid, haploid, and triploid regions in whole genome sequence. (**A**) Expected distribution of all positions in a diploid genome. (**B**) Expected distribution of all positions in a hemizygous genome. (**C**) Expected distribution of all positions in a triploid genome.

With the rapid implementation of HTS in molecular diagnostics and research, there has been a proliferation of  tools for variant identification. There are currently over 80 software packages designed to identify CNVs from WGS alone^14^. These tools predominantly rely on four characteristics of the sequence data: read depth, split reads, read pairs, and assembly-based techniques (reviewed by Zhao, *et al*.^15^). As yet underutilised, the allele balance of reads at a position contributes additional data that can also be exploited for CNV variant detection and validation. This ‘read balance’ is computed from relative read coverage of each allele at a given locus. The read balance can provide information regarding the copy number over the region in the form of the allele-specific copy number (ASCN). Positions in diploid regions of the genome are primarily invariant (homozygous) (as demonstrated in Fig. 1A). This is represented by a relative read distribution peak about 1. The heterozygous positions (SNVs) are represented by a normal distribution centred on 0.5, with the reads split evenly across the two alleles. A deleted (hemizygous or nullizygous) region should not contain any heterozygous positions; nullizygous regions by virtue of not containing genetic information for the aligned region, and hemizygous regions due to containing a single copy of the non-deleted allele, thus resulting in a distribution peak centred around 1, as depicted in Fig. 1B. A triplicated region as represented in Fig. 1C, however, is expected to have homozygous SNVs along with the heterozygous SNVs represented by two normal distributions centred on 0.33 and 0.66, indicating that one third of the reads at a given locus include one allele, and two thirds of the reads include the other.

A number of bioinformatic tools have utilised ASCN for determining CNVs in cancer samples^16–24^. These techniques rely on sequence data from paired tumour and normal tissue samples, and therefore are not suitable for identifying germline CNVs. Alternatively, AS-GENSENG^25^ and ERDS^26^ incorporate read balance information into their algorithms along with read depth based data to discover CNVs. However, there is currently no independent platform providing validation of CNVs using read balance, allowing for integration of this additional data source in established bioinformatic pipelines that use alternative CNV discovery tools. RBV utilises read balance data to validate CNVs identified by other software packages, allowing for prioritisation of CNVs in both research and molecular diagnostic settings.

## Implementation

RBV is a python package, which incorporates the read balance data from positions within the CNV of interest with randomly sampled windows across the genome to predict the authenticity of CNVs. The software extracts the read balance information from a variant call format (vcf) file, uses CNV coordinates from an interval list, and can be employed for both WGS and WES generated data. The analyses can be refined by restricting investigation to callable regions or outside of known gaps in the reference through the inclusion of either an interval list of callable regions, or an interval list of gaps in the reference genome provided by the user. The user can alter the specificity of RBV results through adjusting the parameters: quality and depth cut-offs at each position in the vcf, readbal cut-off for deletion analyses, and the number of randomly generated permutations for the positions and windows. RBV can incorporate data derived from popular variant callers (HaplotypeCaller^27^, SAMtools^28^, Freebayes^29^, and Platypus^30^), and all aligners. However, issues with read balance calculations may arise from non-uniquely aligned regions of the genome if the aligner of choice places these reads at more than one position in the genome, or regions with non-uniform alignment. We therefore recommend using aligners that randomly place reads to only one mappable location by default, such as BWA^31^, and removing regions with low mappability and low sequence complexity by including an intervals file such as that provided in the GATK resources bundle^27^.

RBV is freely available via https://github.com/whitneywhitford/RBV.

## Results

The analysis performed by RBV validates two separate hypotheses: that the putative CNV is a deletion with the region being hemizygous or nullizygous, or that the putative CNV is multiplicated where the region is triploid or greater.

### Deletion analyses

Deletions should represent areas of absence of heterozygosity (AOH), therefore the probability that a deletion exists (p-value) is calculated based on an empirical cumulative distribution function (eCDF). For this calculation, a large number of diploid windows (default 1,000) of the same number of callable base pairs as the CNV of interest are randomly generated from callable regions (if specified by the user) within the individual’s genome, and the number of heterozygous SNVs in each window is subsequently calculated. The empirical p-value is calculated using the eCDF (Eq. 1) for the resulting distribution, with the probability being the proportion of randomly generated windows containing the same number or fewer heterozygous SNVs for the CNV in question.

1 $$Deletion\,p \mbox{-} value=\frac{1}{n}\sum _{i=1}^{n}{1}_{{{\rm{x}}}_{i}\le t}$$

where *x*_1_, *…*, *x*_*n*_ represent the number of heterozygous SNVs within each randomly selected window in the eCDF equation where *n* is the number of randomly generated windows of the same size as the CNV, and t is the number of heterozygous SNVs within the CNV of interest.

### Multiplication analyses

**Figure 2:**
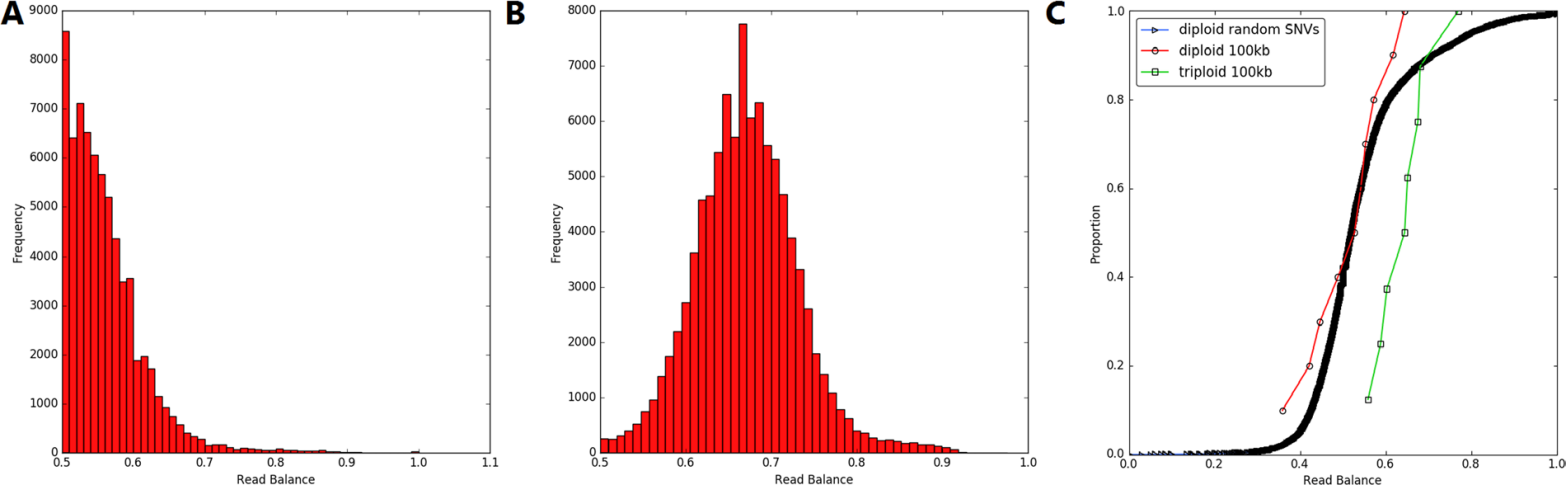
RBV data analysis curves. (**A**) Read balance of the most common allele from heterozygous positions in a diploid genome. (**B**) Read balance of the most common allele from heterozygous positions in a triploid genome. (**C**) CDF curve utilised in a 2-sample KS test, comparing distribution of read balance between randomly generated heterozygous SNVs throughout the reference diploid genome: a 100 kb diploid region, and a 100 kb triplicated region.

The multiplication hypothesis is interrogated using the two-sample Kolmogorov–Smirnov (KS) test. For this analysis we only consider the most common allele at each heterozygous position, which gives the distribution demonstrated in Fig. 2A,B. The differences in the distribution of read balance for randomly generated diploid heterozygous SNVs and the heterozygous SNVs (default 10,000) in the putative CNV are compared using the two-sample KS test, represented in Fig. 2C.

### Performance

**Figure 3:**
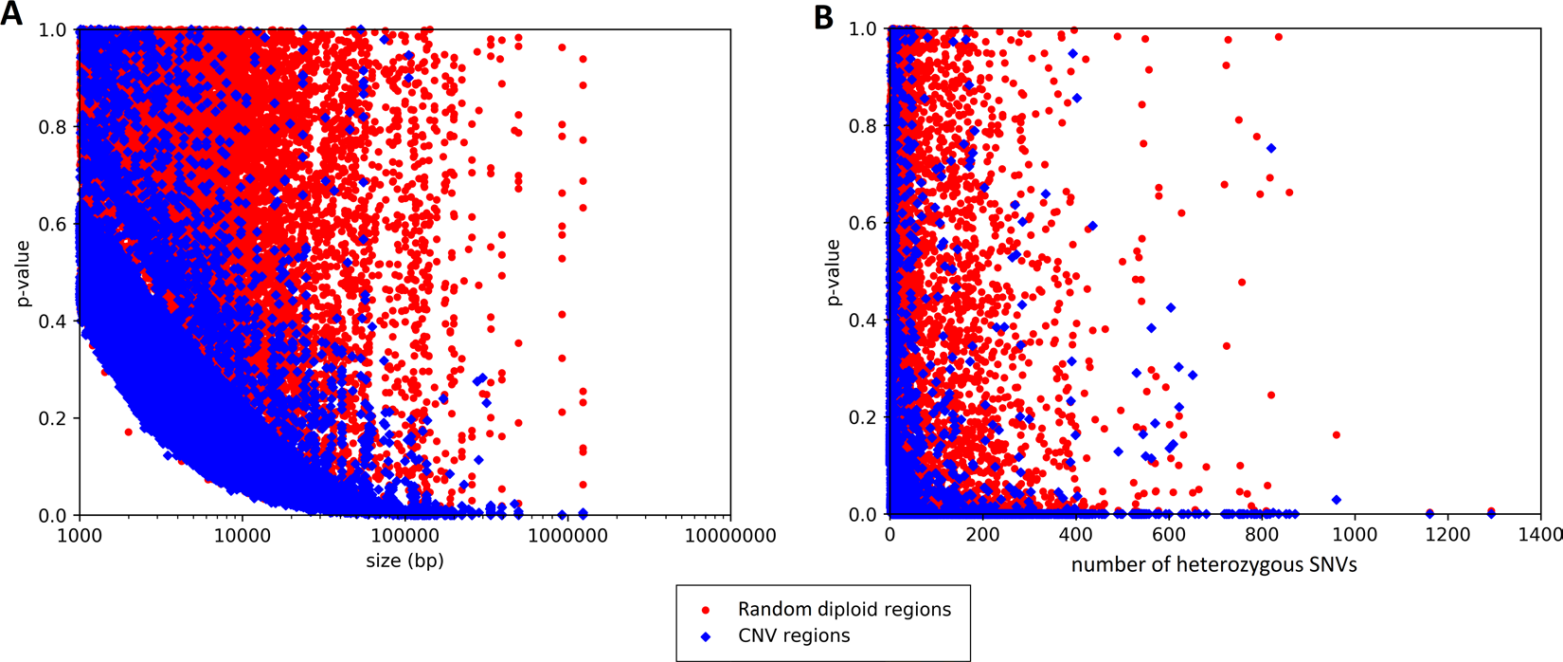
Ability of RBV to prioritise authentic CNVs. Comparision between the results from 31,791 CNV from 25 Phase 3 1000 Genomes Project individuals^39^ and randomly generated diploid regions with the same number of callalble positions as each deletion, or number of heterozygous positions for each duplication. (**A**) Performance of RBV for 23,851 deletions. (**B**) Performance of RBV for 7,940 duplications.

To analyse the performance of RBV, 25 high coverage whole genome alignments and their associated CNV calls >1 kb from Phase 3 of the 1000 Genomes Project^32^ were downloaded. In order to facilitate comparison, diploid regions were randomly generated covering the same number of callable positions^27^ for each deletion and the same number of heterozygous SNVs for each duplication. For the 31,791 CNVs (23,851 deletions, and 7,940 duplications) analysed, RBV was able to identify statistically significant (P ≥ 0.05) CNVs with an overall sensitivity of 6.1% and 41.4% along with a specificity of 99.7% and 47.9%, for deletions and duplications, respectively (Tables 1 and 2). The ability of RBV to prioritise CNVs over the randomly generated regions is demonstrated in Fig. 3.

**Table 1:** RBV performance analysis for deletions for 25 Phase 3 1000 Genomes Project individuals with CNV calls and high coverage whole genome sequence.

Size	Total	TP	FN	TN	FP	Sensitivity	Specificity
>10 kb	3783	1459	2324	3703	80	0.3856727	0.978853
>20 kb	1914	1254	660	1846	68	0.6551724	0.964472
>30 kb	1326	1089	237	1271	55	0.821267	0.958522
>50 kb	738	643	95	702	36	0.8712737	0.95122
>100 kb	397	374	23	375	22	0.9420655	0.944584
>150 kb	169	162	7	161	8	0.9585799	0.952663
>200 kb	93	88	5	89	4	0.9462366	0.956989
>300 kb	55	54	1	52	3	0.9818182	0.945455
>400 kb	31	31	0	30	1	1	0.967742
>500 kb	20	20	0	20	0	1	1
>1 Mb	10	10	0	10	0	1	1
All	23851	1459	22392	23771	80	0.0611714	0.996646

SNV: single nucleotide variant, TP: true positive, FN: false negative, TN: true negative, FP: false positive.

**Table 2:** RBV performance analysis for duplications for 25 Phase 3 1000 Genomes Project individuals with CNV calls and high coverage whole genome sequence.

Number of heterozygous SNVs	Total	TP	FN	TN	FP	Sensitivity	Specificity
1–2	703	126	577	652	51	0.179232	0.927453
3–9	714	434	280	627	87	0.607843	0.878151
10–19	452	341	111	393	59	0.754425	0.869469
20–49	784	640	144	665	119	0.816327	0.848214
50–99	643	581	62	527	116	0.903577	0.819595
100–199	695	639	56	551	144	0.919424	0.792805
200–499	489	460	29	346	143	0.940695	0.707566
500+	82	69	13	45	37	0.841463	0.548780
All	7940	3290	4650	3806	4134	0.414358	0.479345

SNV: single nucleotide variant, TP: true positive, FN: false negative, TN: true negative, FP: false positive.

The comparison shows a separation between CNVs and random regions, with an enrichment of CNVs with low p-values. The enrichment is increasingly marked for CNVs of greater size or greater number of heterozygous SNVs. Therefore, RBV has reduced sensitivity to detect smaller CNVs (<30 kb for deletions and <20 heterozygous SNVs for duplications) due to the reliance upon relatively infrequent heterozygous positions in the randomly generated windows for deletion analysis, and the increased power of a 2-sample KS test with a greater number of heterozygous positions in the CNV. These inherent biases are responsible for the poor overall sensitivity and specificity, and we recommend using RBV for prioritising deletions >30 kb (82.1% sensitivity and 95.9% specificity) and duplications with at least 20 heterozygous SNVs (81.6% sensitivity and 84.8% specificity).

In order to determine the performance of RBV based on the number of random windows sampled per deletion (used to calculate the eCDF), a power analysis was performed using a subset of six of the 1000 Genomes Phase 3 individuals from different populations, consisting of 1,358 deletions in total. RBV was run using 100, 500, 1000 (default), 5000 and 10000 random window permutations per deletion with analyses separated into six bin sizes (1–10 kb, 10–50 kb, 50–100 kb, 100–500 kb, 500 kb-1 Mb, and 1 Mb+). Sensitivity and specificity (P ≥ 0.05 deletion vs. random diploid) was consistent for all bin sizes for random window permutations 500 and greater. The size of deletion had a far greater effect on the ability of RBV to sensitively and accurately identify deletions than random window permutations, where deletions 1–10 kb had a sensitivity of 0%, 10–50 kb had a sensitivity of 13.2%, 50–100 kb had a sensitivity of 84%, and deletions >100 kb had a sensitivity of 100%. Full analyses are presented in the Supplementary Data.

### Use cases

We established the ability of RBV to correctly identify CNVs in the context of causative mutations using two use cases. Firstly, our in-house CNV filtering and prioritisation pipeline (including RBV) was applied to WGS reads from two siblings who presented with recurrence of ataxia, deafness, developmental delay, rhabdomyolysis, cardiomyopathy and hypothyroidism^33^. The pipeline identified a 34 kb deletion encompassing exons three to nine (NC_000022.10:g.20028959_20062955del33997) resulting in nullizygosity over this region in both siblings. Using default parameters and the GATK callable intervals file, RBV validated the presence of this CNV in both siblings when compared to other regions in the genome of the same size resulting in p-values of p = 0.043 and p = 0.027, with no heterozygous SNVs present across the deleted region for both siblings.

Another use for RBV is to test the potential inheritance of CNVs. Using HTS our laboratory recently identified a causative heterozygous 19.6 Mb 2q37 terminal deletion (GRCh37 Chr2:233834098–253404903; NC_000002.11:g. 233834098_253404903del) in a child with ASD^34^. There were both WES and WGS data available for the affected child, and WES data for the parents. RBV was run with default parameters and the GATK callable intervals file, for all four sequence sources. RBV confirmed the deletion with p-value = 0.0 from variants called from both WES and WGS from the affected child (with 0 and 92 heterozygous SNVs out of a total 2,930 and 58,396 variants called in the vcf file over the region, respectively). In comparison, the two parents had 284 and 294 heterozygous SNVs in the exonic sequence in the same region (out of 3,089 and 3,154 total variants called), resulting in p-values of 0.898 and 0.936, respectively. Thus RBV provided evidence that the causative deletion was absent in the parents and is therefore *de novo*, confirmed by Sanger sequencing.

## Discussion

As more research and diagnostic centres investigate the identification of CNVs through sequence data, there is increasing need for the ability to prioritise clinically relevant variants called from CNV detection software platforms. Although a number of detection tools use read depth, split reads, read pairs, and assembly-based techniques, the utility of read balance in CNV analysis has so far been largely underutilised. Thus, RBV was developed to exploit this additional piece of sequence information to reinforce calls from CNV calling pipelines, allowing for prioritisation of variants in the identification of pathogenic CNVs when used in conjunction with functional annotations.

We compared the results of RBV from 31,791 CNVs and randomly generated diploid regions. From this we were able to display the ability of RBV to differentiate genuine deletions >30 kb and duplications with >20 heterozygous SNVs from diploid loci. Thus, this software has utility in prioritising putatively pathogenic deletions >30 kb and duplications with >20 heterozygous SNVs. However, the sensitivity and specificity of RBV decreases for smaller variants with fewer heterozygous SNVs.

One limitation of the analyses performed by RBV results from the tendency of CNV breakpoints to occur as a result of replication errors within fragile sites or other repetitive elements^35–37^. Due to the low sequence complexity of such elements, these regions can be problematic for alignment and variant calling algorithms, resulting in low confidence SNV calls which are often excluded from callable intervals files. As such, if a callable intervals file is included, the search space for CNVs will be reduced, decreasing the ability of the software to sensitively identify true CNVs. Without the inclusion of a callable intervals file, variant callers will have a reduced accuracy in calling SNVs, which will subsequently result in a decreased ability of RBV to sensitively identify true duplications and potentially decrease the specificity for deletions. Thus, the propensity for CNV breakpoints to occur within repetitive regions is potentially partially responsible for RBVs performance bias for larger CNVs.

We were also able to demonstrate the execution of RBV using three clinical cases (two families), including successful identification of a 34 kb causative deletion from WGS, and the identification of a 19.6 Mb deletion from WGS and WES, along with confirmation of mode of inheritance.

## Conclusions

RBV is a software tool designed to assist in the rapidly expanding speciality of identifying clinically relevant CNVs through prioritisation.

The software includes utility for both multiplication and deletion analysis of nominated CNV sites from both WES and WGS data. Sample data for the operation of RBV is available via the GitHub repository.

### Ethics approval and consent to participate

The 1000 Genomes Project Phase 3 data was obtained directly from the The International Genome Sample Resource made available under the Fort Lauderdale Agreement^38^.

The genetic analysis and de-identified publication of variants for use cases was performed under the approval of the New Zealand Northern B Health and Disability Ethics Committee (12/NTB/59) in accordance with guidelines and regulations in the Ethical Guidelines for Observational Studies from the New Zealand National Ethics Advisory Committee. Parents provided written informed consent.

### Availability and requirements

Project name: RBV. Project home page: https://github.com/whitneywhitford/RBV. Operating system(s): Linux.Programming language: Python 2.7. Other requirements: SAMtools 1.3 or higher, tabix. License: GPL v3. Any restrictions to use by non-academics: None.

## Supplementary information

Supplementary Information

## Data Availability

The datasets analysed for the performance of RBV are available via request.
