# Supplementary material for: RBV: Read balance validator, a tool for prioritising copy number variations in germline conditions
Source: Sci Rep. 2019 Nov 15;9:16934. doi: 10.1038/s41598-019-53181-7 (PMC6858463; doi:10.1038/s41598-019-53181-7)
Supplement: Supplementary file 1 — Supplementary Information [file 41598_2019_53181_MOESM1_ESM.docx]

**RBV: Read balance validator, a tool for prioritising copy number variations in germline conditions**

Whitney Whitford^1,2^*, Klaus Lehnert^1,2^, Russell G. Snell^1,2^, Jessie C. Jacobsen^1,2^

1 School of Biological Sciences, The University of Auckland, New Zealand

2 Centre for Brain Research, The University of Auckland, New Zealand

*corresponding author

email: [whitney.whitford@auckland.ac.nz](mailto:whitney.whitford@auckland.ac.nz)

**Supplemental Figures and Tables**

**Power Analysis**

A power analysis for the number of random window permutations for deletions of multiple sizes was performed using deletions >1kb from six individuals of a mixture of ethnicities from the 1000 Genomes Project Phase 3^32^ (HG01879 – African Caribbeans in Barbados [ACB], NA19238 – Yoruba in Ibadan Nigeria [YRI], HG00419 – Southern Han Chinese [CHS], HG00268 – Finnish in Finland [FIN], NA12878 – Utah Residents (CEPH) with Northern and Western European Ancestry [CEU], and HG00096 – British in England and Scotland [GBR]). RBV was run using 100, 500, 1000 (default), 5000 and 10000 random window permutations per deletion with performance analyses separated into six bin sizes: 1-10 kb, 10-50 kb, 50-100 kb, 100-500 kb, 500 kb-1 Mb, and 1 Mb.

**Supplementary table 1.** Summary of RBV power analysis based on deletions from six 1000 Genomes Project individuals

| **Sensitivity** | | | | | |
| --- | --- | --- | --- | --- | --- |
|  | Number of window permutations | | | | |
| Size | 100 | 500 | 1000 | 5000 | 10000 |
| 1-10kb | 0 | 0 | 0 | 0 | 0 |
| 10-50kb | 0.132 | 0.167 | 0. 167 | 0. 167 | 0. 167 |
| 50-100kb | 0.84 | 0.84 | 0.84 | 0.84 | 0.84 |
| 100-500kb | 1 | 1 | 1 | 1 | 1 |
| 500kb-1Mb | 1 | 1 | 1 | 1 | 1 |
| 1Mb+ | 1 | 1 | 1 | 1 | 1 |
|  |  |  |  |  |  |
| **Specificity** | | | | | |
|  | Number of window permutations | | | | |
| Size | 100 | 500 | 1000 | 5000 | 10000 |
| 1-10kb | 1 | 1 | 1 | 1 | 1 |
| 10-50kb | 0.995 | 0.990 | 0.990 | 0.990 | 0.990 |
| 50-100kb | 1 | 0.96 | 0.96 | 0.96 | 0.96 |
| 100-500kb | 0.909 | 0.909 | 0.909 | 0.939 | 0.939 |
| 500kb-1Mb | 1 | 1 | 1 | 1 | 1 |
| 1Mb+ | 1 | 1 | 1 | 1 | 1 |

**Supplementary table 2.** RBV performance analysis for deletions from six 1000 Genomes Project individuals with 100 random window permutations

| **Size** | **Total** | **TP** | **FN** | **TN** | **FP** | **Sensitivity** | **Specificity** |
| --- | --- | --- | --- | --- | --- | --- | --- |
| 1-10kb | 1094 | 0 | 1094 | 1094 | 0 | 0 | 1 |
| 10-50kb | 204 | 27 | 177 | 203 | 1 | 0.132 | 0.995 |
| 50-100kb | 25 | 21 | 4 | 25 | 0 | 0.84 | 1 |
| 100-500kb | 33 | 33 | 0 | 30 | 3 | 1 | 0.909 |
| 500kb-1Mb | 1 | 1 | 0 | 1 | 0 | 1 | 1 |
| 1Mb+ | 1 | 1 | 0 | 1 | 0 | 1 | 1 |

SNV: single nucleotide variant, TP: true positive, FN: false negative, TN: true negative, FP: false positive

**Supplementary table 3.** RBV performance analysis for deletions from six 1000 Genomes Project individuals with 500 random window permutations

| **Size** | **Total** | **TP** | **FN** | **TN** | **FP** | **Sensitivity** | **Specificity** |
| --- | --- | --- | --- | --- | --- | --- | --- |
| 1-10kb | 1094 | 0 | 1094 | 1094 | 0 | 0 | 1 |
| 10-50kb | 204 | 34 | 170 | 202 | 2 | 0.167 | 0.990 |
| 50-100kb | 25 | 21 | 4 | 24 | 1 | 0.84 | 0.96 |
| 100-500kb | 33 | 33 | 0 | 30 | 3 | 1 | 0.909 |
| 500kb-1Mb | 1 | 1 | 0 | 1 | 0 | 1 | 1 |
| 1Mb+ | 1 | 1 | 0 | 1 | 0 | 1 | 1 |

SNV: single nucleotide variant, TP: true positive, FN: false negative, TN: true negative, FP: false positive

**Supplementary table 4.** RBV performance analysis for deletions from six 1000 Genomes Project individuals with 1,000 random window permutations (default)

| **Size** | **Total** | **TP** | **FN** | **TN** | **FP** | **Sensitivity** | **Specificity** |
| --- | --- | --- | --- | --- | --- | --- | --- |
| 1-10kb | 1094 | 0 | 1094 | 1094 | 0 | 0 | 1 |
| 10-50kb | 204 | 34 | 170 | 202 | 2 | 0.166667 | 0.990 |
| 50-100kb | 25 | 21 | 4 | 24 | 1 | 0.84 | 0.96 |
| 100-500kb | 33 | 33 | 0 | 30 | 3 | 1 | 0.909 |
| 500kb-1Mb | 1 | 1 | 0 | 1 | 0 | 1 | 1 |
| 1Mb+ | 1 | 1 | 0 | 1 | 0 | 1 | 1 |

SNV: single nucleotide variant, TP: true positive, FN: false negative, TN: true negative, FP: false positive

**Supplementary table 5.** RBV performance analysis for deletions from six 1000 Genomes Project individuals with 5,000 random window permutations

| **Size** | **Total** | **TP** | **FN** | **TN** | **FP** | **Sensitivity** | **Specificity** |
| --- | --- | --- | --- | --- | --- | --- | --- |
| 1-10kb | 1094 | 0 | 1094 | 1094 | 0 | 0 | 1 |
| 10-50kb | 204 | 34 | 170 | 202 | 2 | 0.166667 | 0.990 |
| 50-100kb | 25 | 21 | 4 | 24 | 1 | 0.84 | 0.96 |
| 100-500kb | 33 | 33 | 0 | 31 | 2 | 1 | 0.939 |
| 500kb-1Mb | 1 | 1 | 0 | 1 | 0 | 1 | 1 |
| 1Mb+ | 1 | 1 | 0 | 1 | 0 | 1 | 1 |

SNV: single nucleotide variant, TP: true positive, FN: false negative, TN: true negative, FP: false positive

**Supplementary table 6.** RBV performance analysis for deletions from six 1000 Genomes Project individuals with 10,000 random window permutations

| **Size** | **Total** | **TP** | **FN** | **TN** | **FP** | **Sensitivity** | **Specificity** |
| --- | --- | --- | --- | --- | --- | --- | --- |
| 1-10kb | 1094 | 0 | 1094 | 1094 | 0 | 0 | 1 |
| 10-50kb | 204 | 34 | 170 | 202 | 2 | 0.167 | 0.990 |
| 50-100kb | 25 | 21 | 4 | 24 | 1 | 0.84 | 0.96 |
| 100-500kb | 33 | 33 | 0 | 31 | 2 | 1 | 0.939 |
| 500kb-1Mb | 1 | 1 | 0 | 1 | 0 | 1 | 1 |
| 1Mb+ | 1 | 1 | 0 | 1 | 0 | 1 | 1 |

SNV: single nucleotide variant, TP: true positive, FN: false negative, TN: true negative, FP: false positive


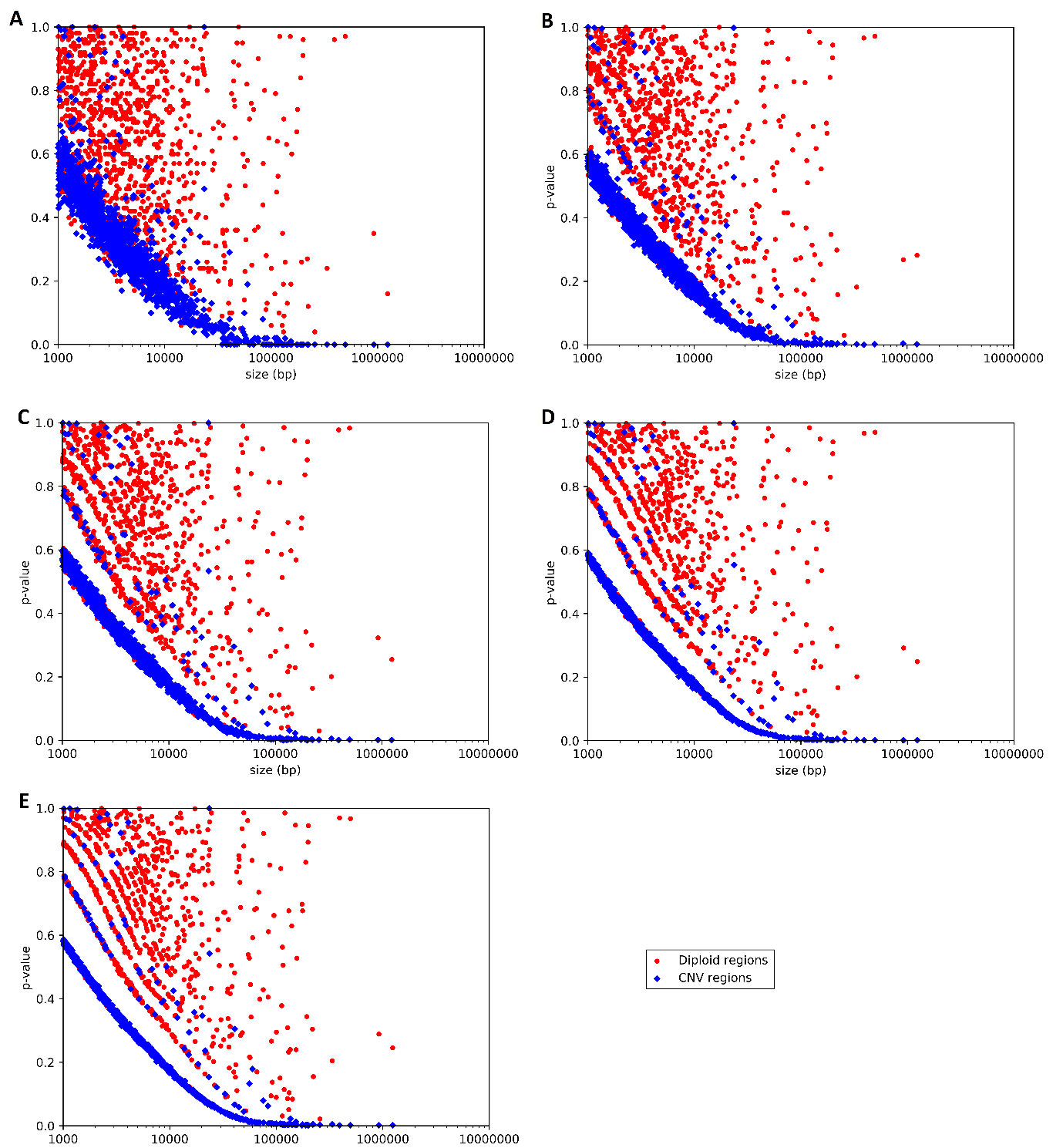


**Supplementary figure 1.** Power analysis based on comparision between the results from 1,358 deletions from six Phase 3 1000 Genomes Project individuals^32^ and differing numbers randomly generated diploid regions of the same number of callalble positions as each deletion. A) 100 random diploid regions per deletion, B) 500 random diploid regions per deletion, C) 1,000 random diploid regions per deletion, D) 5,000 random diploid regions per deletion, E) 10,000 random diploid regions per deletion
